# Microwave-Assisted Saponification Method Followed by Solid-Phase Extraction for the Characterization of Sterols and Dialkyl Ketones in Fats

**DOI:** 10.3390/foods10020445

**Published:** 2021-02-18

**Authors:** Steven Mascrez, Sabine Danthine, Giorgia Purcaro

**Affiliations:** 1 Analytical Chemistry Lab, Gembloux Agro-Bio Tech, University of Liège, 5030 Gembloux, Belgium; steven.mascrez@uliege.be; 2 Department of Food Science and Formulation, Gembloux Agro-Bio Tech, University of Liège, 5030 Gembloux, Belgium; sabine.danthine@uliege.be

**Keywords:** dialkylketones, sterols, microwave-assisted saponification, solid-phase extraction, chemical interesterification, gas chromatography

## Abstract

Unlike other fields, the methods routinely applied for fats and oils are still tied to traditional, time- and solvent-consuming procedures, such as saponification, column chromatography and thin-layer chromatography. In this paper, microwave-assisted saponification followed by a lab-made solid-phase extraction was optimized for the characterization of either dialkyl ketones (DAK) or sterols or both simultaneously. The instrumental determination was performed by gas chromatography- flame ionization detector (GC-FID) for quantification and gas chromatography-mass spectrometry (GC-MS) for confirmation purposes. The proposed method showed good recoveries (>80%) and limit of quantification (0.04–0.07 μg/g for the 4 DAK and of 0.07 μg/g for α-cholestanol). Repeatabilities (*n* = 3) were below 15% for DAKs and generally lower than 6% for sterols. Accuracy on the entire sterol profile was confirmed in comparison to the International Olive Council reference method. The method was finally applied to real-world samples before and after chemical interesterification.

## 1. Introduction

Differently from other fields, such as pharmaceutical, food analysis is highly dependent on a preliminary sample preparation step, mainly the extraction and isolation of the target analytes. Despite the advancement in the field, with the introduction of highly efficient instrumentation, automated sample-preparation workstations and miniaturized techniques, most of the methods routinely applied to food analysis remain tedious, time-consuming and highly manipulative [1,2]. This is particularly true in the field of fats and oil, where still Soxhlet extraction, saponification involving a large amount of solvents, followed by thin-layer chromatography (TLC) or column chromatography, are widely used. 

Among more advanced extraction techniques, pressurized-liquid extraction (PLE) and microwave-assisted extraction (MAE) have gained popularity. MAE is a rapid, solvent saving and efficient extraction technique. It exploits the energy produced by non-ionizing radiations to intimately heat the sample-extractant system in few seconds by inducing dipole rotation and ionic conduction [3]. Despite the substantial advantage that microwave use may provide to most of the temperature-involving process in analytical chemistry, the exploitation of this technique is still limited. MAE has been investigated mainly in the analysis of contaminants in food [1,2]. In contrast, a limited number of applications have exploited the use of MAE to accelerate the saponification step (microwave-assisted saponification, MAS) [4,5,6,7,8,9,10,11].

Regarding the isolation step, despite the widespread use of solid-phase extraction (SPE) also in the lipid and fats field [6,12,13,14], routine methods remain tight to more tedious procedures [15,16]. 

It appears evident that the optimization of a robust and validated procedure for more rapid characterization of oils and fats is necessary. This paper aims to provide a flexible method exploiting MAS to efficiently and rapidly performed the sample saponification, followed by a lab-made SPE to isolate the fraction of interest. In this context, sterols and dialkyl ketones (DAK) have been targeted.

Sterols and, in particular, phytosterols play an important role in the human diet [17,18] and in verifying the authenticity of the oil products (e.g., to discriminate olive oil from seed oil) [19]. The traditional determination of sterols usually involved a first saponification step, followed by liquid-liquid extraction of the unsaponifiable, TLC for isolation of the sterol and final instrumental determination, most often in gas chromatography (GC) after derivatization [20]. Several works proposed the use of SPE as an alternative to TLC [13,14,21,22].

Dialkyl ketones (DAKs) are by-products of the chemical interesterification process, industrially performed to redistribute the fatty acids among triglycerides changing the physicochemical characteristics of fat (e.g., changing crystallization behavior and the melting point, improving plasticity) [23]. The formation of DAKs in chemically interesterified products was presented for the first time by Verhé at the American Oil Chemists’ Society (AOCS) conference in 2006, but never published [24]. So far, only two papers have dealt with DAKs determination in food [25,26]. Both of these works involved a first saponification step followed by a column chromatography purification involving the use of a high amount of solvent (170 mL of hexane/ethyl ether 1%) [25] or a commercially available silica SPE (1 g) using about 25 mL of solvent for the only elution of DAKs. The method of Mariani et al. performed the final determination in GC-FID, confirming the identity in mass spectrometry (MS), while Santoro et al. used either GC-MS or LC-high resolution MS (HRMS) [26].

In this paper, a MAS-SPE method is optimized for the simultaneous or alternative characterization of DAKs and sterols, followed by GC-FID for quantification and GC-MS for confirmation.

## 2. Materials and Methods

### 2.1. Chemicals and Reference Solutions

Diethyl ether, n-hexane, ethyl acetate, pyridine, methanol (analytical grade), potassium hydroxide, sodium methoxide, citric acid, phenolphthalein indicator (1.0%, *m*/*v*), anhydrous Na_2_SO_4_, 2,7-dichlorofluorescein were purchased from MilliporeSigma^®^ (Belfonte, PA, USA). Standards of 9-octadecanone (C10:0-9:0), 11-heneicosanone (C11:0-11:0), 14-heptacosanone (C14:0-14:0), 18-pentatriacontanone (C18:0-18:0), N,O-Bis(trimethylsilyl)trifluoroacetamide (BSTFA), α-cholestanol and betulin were kindly provided by MilliporeSigma^®^. Silica gel (60 Å, 70–230 mesh, 63–200 μm for column chromatography) was from MilliporeSigma. Bleaching earth (Tonsil Optimum 210FF) was from Südchemie.

The optimization of the method was realized with an extra virgin olive oil bought in a local supermarket (Gembloux, Belgium).

### 2.2. Laboratory-Scale Batch Chemical Interesterification

Lab-scale chemical interesterification was performed as reported in [27,28] with minor adjustments. Briefly, the fat blend was neutralized with NaOH whether the free fatty acid content exceeds 0.05% and further dried at 25 mbar at 120 °C for 30 min. The catalyst, sodium methoxide (0.1%, *w*/*w*), was then added and the reaction occurred under vacuum (25 mbar) at 90 °C for 30 min. Afterwards a 20% citric acid solution was added to deactivate the catalyst. The fats were then treated with bleaching earth (1.5%, 25 mbar, 90 °C for 30 min) to remove the color formed during the interesterification reaction. Finally, the bleaching earth was removed by filtration.

The following samples were treated: palm oil, rapeseed oil (raw and hydrogenated), shea butter.

### 2.3. Official Method for Sterol Determination

The analysis was carried out following the method adopted by the International Olive Council (IOC) [29]. Briefly, α-cholestanol was added in a flask (50 mg/kg final concentration) and evaporated until dry. Five g of olive oil and 50 mL of 2 M KOH in ethanol/water (80/20 *v*/*v*) were added to the flask. The solution was boiled for 20 min. After cooling, the extract was transferred in a separatory funnel with the addition of 50 mL of ultrapure water. The unsaponifiable fraction was extracted by liquid-liquid extraction (LLE) with three fractions (80, 60, 60 mL) of Et_2_O. The three fractions were combined and washed repetitively with 50 mL of ultrapure water until neutrality (verified using phenolphthalein as pH-indicator). The step was repeated until the water was colorless. The ether fraction was filtered on anhydrous sodium sulphate to remove the residual water. The fraction was evaporated until dryness in a rotary evaporator. The dried olive oil extract was dissolved in 1.0 mL of n-hexane. 300 μL was deposited on a silica TLC plate, developed using a mixture of n-hexane/diethyl ether 65/35 (*v*/*v*), Sterols and triterpene dialcohols band was scratched and collected for further analysis previous derivatization with 100 μL of BSTFA and 100 μL of pyridine. The reaction occurred at room temperature for at least 15 min. Then, the sample was dried again and dissolved in 1.0 mL of n-hexane for injection.

### 2.4. Microwave-Assisted Saponification

Internal standards (ISs) for DAKs (C14:0-C14:0) and sterols (α-cholestanol) were added to the microwave vessel and dried with a gentle flow of nitrogen. 2.5 g of the sample were weighted in the vessel and 25 mL of KOH 2 M in ethanol/water or KOH 2 M in methanol/water (80/20 *v*/*v*) were added in the microwave vessel. Saponification was carried out in an Ethos X microwave system equipped with a high-pressure SR 12 rotor from Milestone Srl (Milan, Italy). The treatment temperature (90 °C) was reached within 2 min at 800 W and maintained for 10 min, under constant magnetic stirring. After cooling, the unsaponifiable fraction has been extracted following the same proportion between sample and solvent (40, 30, 30 mL) as in LLE mentioned for the reference protocol (IOC). The step was repeated until the water was colorless.

Hexane fraction was filtrated with anhydrous sodium sulphate to remove water. The fraction was evaporated until dryness in a rotary evaporator prior to the following purification step.

### 2.5. Solid-Phase Extraction Isolation of Sterols and Dialkyl Ketones

**Figure 1:**
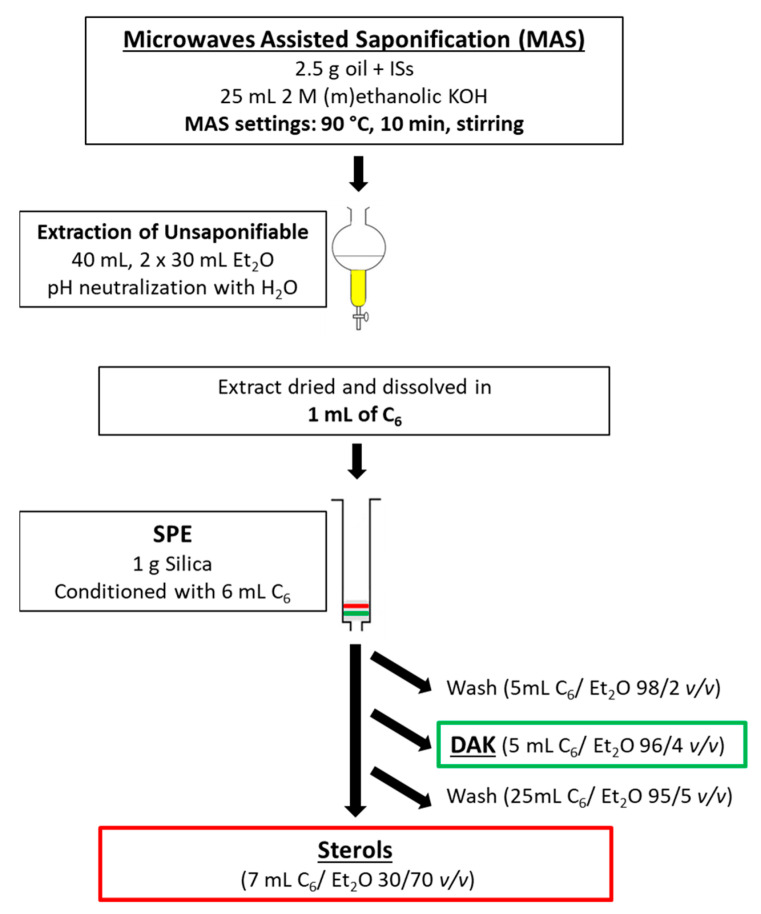
Scheme of the sample preparation procedure.

The final optimized procedure was the following: 1.0 g of silica (60 Å, 70–230 mesh, 63–200 μm for column chromatography) was weighted and put in a 6.0 mL SPE cartridge. The dried olive oil extract was dissolved in 1.0 mL of n-hexane. The SPE was conditioned by 6.0 mL of n-hexane. The sample was loaded into the cartridge. 5.0 mL of n-hexane/diethyl ether 98/2 (*v*/*v*) was used to wash the sample. 5.0 mL of n-hexane/diethyl ether 96/4 (*v*/*v*) was used to collect the dialkyl ketones fraction. 25.0 mL of n-hexane/diethyl ether 95/5 (*v*/*v*) was used as an intermediate wash. 7.0 mL of n-hexane/diethyl ether 30/70 was used to collect the sterols fraction. A scheme of the entire procedure is reported in Figure 1.

Both fractions of interest were evaporated to dryness. Sterols fraction was derivatized with the same procedure as aforementioned. Each fraction was dissolved in 1 mL of n-hexane for analysis.

### 2.6. GC-FID Analysis

GC-FID analyses were performed on a Thermo Fischer Trace Ultra 1300 using a Macherey Nagel (Optima-5-Accent) column, 30 m × 0.25 mm i.d. × 0.25 μm. GC conditions were as follows: injector temperature 300 °C, injection volume 1.0 μL in split conditions with a split ratio 1:10, flow rate of helium career gas 1.3 mL/min. Temperature program: initial temperature 80 °C, ramp to 160 °C at 20 °C/min, and ramp to 340 °C at 5 °C/min hold 1 min. FID conditions: data acquisition 50 Hz, flame temperature 350 °C, air flow 350.0 mL/min, H_2_ flow 35.0 mL/min and makeup gas flow 30.0 mL/min. The DAK and sterols quantifications were performed using the internal standard method. 14-heptacosanone and α-cholestanol, were used for the quantification of DAK and sterols, respectively.

### 2.7. GC-MS Analysis

All analyses were performed on a Shimadzu GCMS-TQ8050 NX, consisting of a GC2030 coupled to a triple-quadrupole mass spectrometer detector (TQ-MS) (Shimadzu, Germany), equipped with an AOC20i autosampler. The chromatographic column was a 30 m × 0.25 mm i.d. × 0.5 μm *d_f_* SLB-5ms capillary column [(silphenylene polymer, practically equivalent in polarity to poly (5% dipheny l/95% methylsiloxane)] kindly obtained from MilliporeSigma (Belfonte, PA, USA). GC oven temperature program: 80 °C to 160 °C at 20 °C/min and to 340 °C at 5 °C/min hold 1 min. Carrier gas: helium in constant linear velocity mode at 35.9 cm/s, corresponding to an initial inlet over-pressure of 45.6 kPa. The MS was operated in single-quadrupole mode, in EI mode at 70 eV. The ion source and transfer line temperatures were 200 °C and 280 °C, respectively. The scan range was set to 50–700 *m*/*z*, with an acquisition frequency of 10 Hz. Data were acquired using Shimadzu GCMSolution ver 4.45 (Shimadzu, Germany). NIST17s MS commercial libraries were used for putative identification.

## 3. Results and Discussion

The SPE optimization was first performed to maximize the throughput during the optimization of the MAS condition. In fact, after each test for the MAS optimization, a purification step is required before the final determination, which was performed by SPE after proving the equivalence with the official TLC procedure.

For the optimization of both MAS and SPE, an olive oil sample was spiked with a mix of DAKs (C10:0-C9:0, C11:0-C11:0, C14:0-C14:0, C18:0-C18:0) and the ISs for sterols (α-cholestanol and betulin). The main advantage of using the latter two standards is that they bracket the entire sterol fraction, assuring the whole elution monitoring their presence.

The goal was to enhance the overall sample throughput minimizing the solvent consumption. The proposed method can be used for the determination of either DAK or sterols or both simultaneously.

During the entire optimization, the results obtained were compared with the profile obtained applying the IOC protocol as the “gold standard method” for the sterols, while the DAKs were constantly monitored using the ISs and the GC-MS data.

### 3.1. Optimization of the SPE Cartridge for the Isolation of DAK and Sterols

For the optimization of the SPE cartridge, the first part of the procedure followed the IOC protocol carefully to add only a variable, namely the SPE purification that replaced the TLC. ISs were added just before loading the sample into the SPE cartridge to monitor only this step. The SPE cartridge was lab-packed using 1 g of column chromatography silica. The elution solvent mixture used was hexane/Et_2_O, but with an increased elution strength to reduce the total volume compared to the previous volume ratio used by Santoro et al. and Mariani et al. (i.e., 99/1 *v*/*v* heptane or hexane/Et_2_O, respectively) [25,26]. Mariani et al. performed a first washing step with 70 mL of hexane and then eluted the DAKs with 180 mL hexane/Et_2_O (99/1 *v*/*v*), using a 15 g silica column [25]. Santoro et al. used a 1 g silica column reducing the solvent volume to 12 mL of heptane for the washing step and 12 mL heptane/Et_2_O (99/1 *v*/*v*) for the collection of DAK [26]. In the present work, the first washing step was performed with 5 mL of hexane/Et_2_O (98/2 *v*/*v*) to remove the presence of alkanes and the DAKs were then eluted with a mixture of hexane/Et_2_O of 96/4 *v*/*v*. The complete elution of the compounds of interest in the 5 mL-fraction was verified by the absence of DAKs in the previous and following fractions (evaluated by GC-MS) and by calculating the recoveries of the 4 added DAKs, which resulted all above 80%.

**Figure 2:**
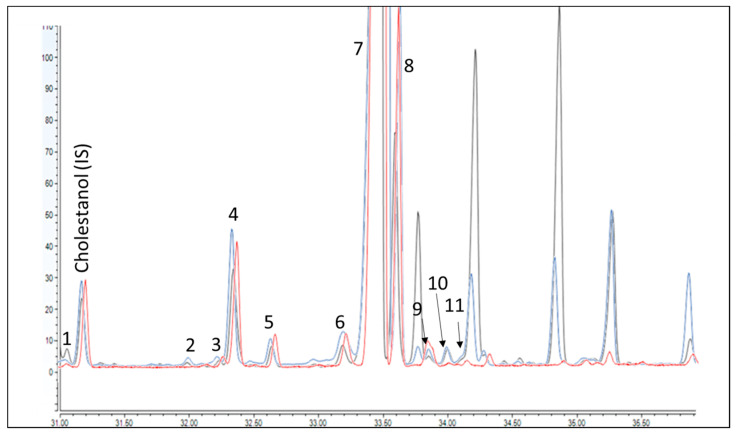
GC-FID trace chromatogram of the sterol fraction obtained using the IOC method (red trace), the first optimized method where the sterol fraction was eluted after 5 mL of hexane/Et_2_O (88/12, *v*/*v*) (black trace) and the final conditions (blue trace). Identification as for Table 1.

Once optimized and assured that the DAK fraction was completely and repeatedly eluted in the 5 mL fraction, the elution of the sterol fraction was optimized. A first trial was done by increasing the elution strength significantly to reduce the intermediate washing step between the DAKs and the sterols [13,14,30]. The eluting fractions were tested by collecting small portions of volume (i.e., 2 mL) and verifying the content in GC-FID. At the first stage, an intermediate washing step of 5 mL of hexane/Et_2_O (88/12 *v*/*v*) was optimized, before elution of the sterols with 7 mL of a mixture of hexane/Et2O (30/70 *v*/*v*). However, using this condition, three intense peaks, not present in the sterol profile obtained with the IOC method, eluted in the sterol fraction (Figure 2). They were identified as 4,4’-dimethylsterols (i.e., cyclartenol (a), 24-methylencycloartenol (b) and cycloartanol (c)) by GC-MS. These compounds are usually considered apart from the sterols (4-desmethylsterols) since they can coelute with sterols of interest. Therefore, the strength of the intermediate washing was gradually reduced with the attempt to separate these compounds. Unfortunately, this determined the use of a much higher volume of solvents, heading to a washing step of 25 mL of hexane/Et_2_O (95/5, *v*/*v*) to reduce the interfering peaks to ~30% of the original value, at the expense of a loss of 1% of ß-sitosterol (the most abundant compound) in the washing step. This compromise was considered sufficient to do not completely hindered the quantification of the peaks coeluted nearby. The repeatability of the SPE step and the recovery were assessed on the standards providing value on the 85–115% range for DAKs and 94% for α-cholestanol and 85% for betulin.

### 3.2. Optimization of the Microwave-Assisted Saponification

Microwave-assisted saponification (MAS) was optimized to increase the overall sample throughput. In fact, microwave not only allows for a more efficient heating step but can also treat more samples simultaneously (12 in our system). A first attempt was made performing the saponification at 60 °C for 20 min, but the process resulted in an excessive residual fat. Therefore, the temperature was increased to keep a short process time (10 and 20 min were tested). Moreover, the efficiency of ethanolic and methanolic KOH solution was compared. Indeed, although ethanol is considered a green solvent, its cost is fluctuant, particularly in relation to the Covid-19 crisis that also affected its availability in the short term. Therefore, methanol, which has similar properties, can be a valid alternative.

**Figure 3:**
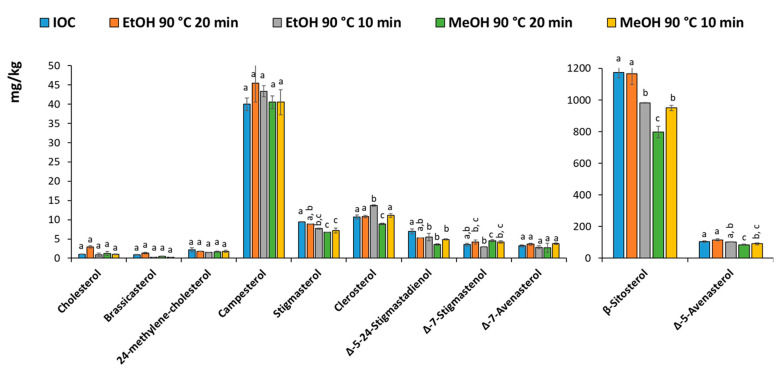
Barplot of the sterols using the IOC method or the different MAS saponification followed by SPE. Same letter indicates no significant difference.

Figure 3 shows the profiles of the sterols quantified using the IOC method (conventional saponification and TLC or the MAS followed by SPE purification. The results were generally in agreement, although slightly lower results can be observed using MeOH for 20 min. It can be concluded that ethanol and methanol can be used alternatively, however, considering the low availability and high cost of ethanol during the period that this work was carried out, methanol was chosen along with a treatment time of 10 min.

The sterol profile obtained with the proposed method was compared to the one obtained by applying the IOC protocol. Data is reported in Table 1. The results were in good agreement. The proposed MAS-SPE method provided a better repeatability (*n* = 3), showing coefficients of variation (CV%) <3% for all the compounds, except Δ5-avenasterol for which a CV% of 5.8% was obtained. 

### 3.3. Method Performance

The limit of quantification (LOQ) was calculated from the signal-to-noise ratio (S/N) on the olive oil spiked with the DAKs and sterol standards used for the optimization of the method. Value of 0.04–0.07 μg/g for the 4 DAK and of 0.07 μg/g for α-cholestanol and 0.14 μg/g for betulin were obtained. These LOQ fulfill the European Regulation requirements to detect a limit of 1 mg/g of brassicasterol in olive oils [EU Reg 2015/1830].

The recoveries (*n* = 3) of the entire method were above 80% for DAKs and 108% for α-cholestanol and 82% for botulin. Repeatabilities (*n* = 3) were below 15% for DAKs and generally lower than 6% for sterols (Table 1). Accuracy, evaluated in comparison to the IOC reference method, was satisfactory (Table 1).

Table 2 reports the proposed method compared to others reported in the literature in terms of Analytical Eco-Scale [31]. The latter system proposed a way to assess the greenness of an analytical procedure. Although we acknowledge that we cannot claim to offer a green approach, the reduction in solvent and manipulation remains significant. Therefore, the use of the Eco-Scale was chosen as a standardized method to evaluate the analytical procedures. Nevertheless, the Eco-Scale system has some limitations, for instance, the penalty assigned on the quantity of solvent used is divided into three ranges, namely <10 mL, 10–100 mL, and >100 mL, therefore, some differences are flattened.

Considering the common saponification procedure, used both in the few methods proposed for DAK analysis as for sterol determination, the method here presented is midway in the solvent consumption (i.e., 125 mL). However, it is worth mention that the methods reporting the lowest solvent amounts (i.e., 29.5 and 62 mL) use an additional SPE cartridge for the unsaponifiable extraction [13,14].

Comparing the method for DAK that we proposed with the only other two reported in the literature [25,26], our method use about half of the solvent for the saponification (saponification and unsaponifiable extraction), i.e., 125 mL vs. 250 mL. The purification involved a 1 g SPE in the method here proposed as well as in the method proposed by Santoro et al. [26], but the solvent consumption was reduced by about 2.5 times.

Compared to the methods proposed for sterols analysis, the method here proposed used the lowest quantity of solvent regarding the purification step, i.e., 42 mL. Moreover, it is important to stress that part of this volume can be used to analyze DAK, so with a volume similar to the one reported in [13], two classes of compounds can be determined.

### 3.4. Real-World Samples

The proposed MAS-SPE method was applied to a series of real-world samples, namely palm oil, rapeseed oil (raw and hydrogenated), shea butter, and a mixture of the formers after chemical interesterification (CIE). All the samples were analyzed in GC-FID for quantification and on GC-MS to verify the fraction eluted from the SPE with fats matrices different from olive oil. All the samples confirmed the robustness of the proposed method. The data on both DAKs and sterols is reported in Table 3. Samples of palm and rapeseed oils before and after CIE were analyzed in triplicate. All the others in single.

**Figure 4:**
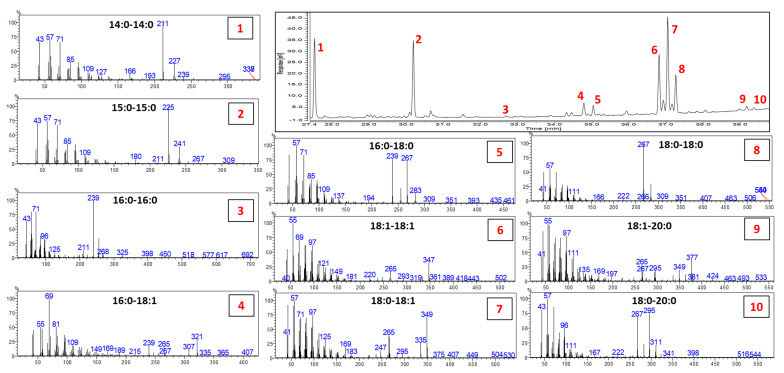
GC trace of the chemically interesterified rapeseed oil (60/40 raw and hydrogenated, respectively). The MS spectra of each of the identified peak are reported labeled with the same number of the corresponding peak and reporting the identification of the DAK.

Since rarely investigated, the DAK profile was studied using GC-MS before quantification of the GC-FID data. Identification of the different DAKs was not possible based only on the NIST Database; therefore, additional information was obtained by a careful evaluation of the MS fragmentation and by the MS data and elution order reported in the few references available [24,25,26]. Figure 4 depicts the profile of the chemically interesterified rapeseed oil (60/40 raw and hydrogenated, respectively), along with the MS spectra of all the DAKs detected. The fragmentation of DAKs in EI-MS is highly characteristic, allowing them to be easily distinguished from other unsaponifiable components. A detailed explanation on the fragmentation mechanism is reported in [26]. In the case of saturated DAKs, the MS spectrum presents fragments deriving from the lateral chains as the predominant fragments, 16 u apart. For instance, the MS spectrum of C18:0-18:0 is characterized by the ion fragments 267 *m*/*z* and 283 *m*/*z*. The two fragments derived from the α-cleavage and the McLafferty rearrangement, respectively. When unsaturation is present, less intense characteristic fragments are detected but still diagnostic. For more details on the pathway of fragmentation, the readers are directed to [25,26]. 

It can be observed from the results reported in Table 3 that the chemical interesterification process caused the formation of DAKs, as expected from the literature. Two samples were available before and after the CIE process; therefore, they were analyzed for both DAKs and sterols to investigate the possible modification of the sterol fraction during this process. So far, only one study has investigated the potential effect of CIE on sterols [30]. The data here reported confirmed the previous finding that the sterol fraction is not affected by the CIE process. Therefore, DAKs are, at present, the only reliable candidate to verify whether the oil and fat underwent such a process. 

## 4. Conclusions

The optimized MAS-SPE method proposed a single solution for the characterization of DAKs and sterols in a single sample preparation step. The method proposed can also be used for the determination of DAKs and sterol independently, providing advantages in terms of solvent-consumption compared to the methods present in the literature, as highlighted in Section 3.3 and Table 2. The solvent consumption, mainly used for the unsaponifiable extraction, could be further reduced by coupling the MAS with the SPE extraction procedure proposed by others [13,14]. Nevertheless, the reduction compared to other more traditional methods is already significant (about half). On the other side, the SPE proposed to isolate sterols and DAK proved very efficient and reduced the solvent consumption compared to any other method reported before.

The outcomes of the method were verified continuously by analyzing the results in both GC-FID and MS for quantitative and qualitative confirmation. Finally, the use of GC-FID for quantification provides a reliable and affordable method for DAKs determination.

## Figures and Tables

**Table 1:** Sterol profile in mg/kg obtained using the IOC protocol (*n* = 3) and the proposed MAS-SPE method (*n* = 3).

Compounds		IOC		MAS-SPE	
		mg/kg	%CV	mg/kg	%CV
1	Cholesterol	0.1	6.8	0.1	0.3
2	Brassicasterol *	0.1	11.2	0.0	0.1
3	24-methylene-cholesterol	0.2	22.8	0.2	0.4
4	Campesterol	4.1	4.1	4.3	2.7
5	Stigmasterol	0.9	1.5	0.7	0.5
6	Clerosterol	1.1	5.2	1.0	0.7
7	β-Sitosterol	118.0	2.7	95.0	1.8
8	Δ-5-Avenasterol *	10.6	4.0	9.3	5.8
9	Δ-5-24-Stigmastadienol	0.7	8.8	0.4	0.4
10	Δ-7-Stigmastenol	0.4	10.1	0.5	0.3
11	Δ-7-Avenasterol	0.3	7.8	0.4	0.4

* indicate 0.01 < *p* < 0.05.

**Table 2:** Comparison of the proposed method vs. other methods reported in the literature based on the Eco-Scale system [31].

	Eco-Scale	Ref
DAK	67	[26]
	69	[25]
Sterols	69	[13]
	59	[21]
	67	[22]
	69	[14]
	66	[15]
DAK/Sterols	75 (DAK)	this method
	71 (DAK + Sterols)	

**Table 3:** DAK and sterol composition of no chemically interesterified (NCIE) and chemically interesterified (CIE) palm oil, NCIE and CIE rapeseed oil, CIE shea butter, CIE mix of raw and hydrogenated rapeseed oil (70/30 and 60/40), CIE mix of rapeseed and palm oil.

Compounds	NCIE Palm Oil	CIE Palm Oil	NCIE Rapeseed Oil (Raw)	CIE Rapeseed Oil (Raw)	CIE 1	CIE 2	CIE 3	CIE 4
**DAK (mg/g)**								
C15:0-C15:0	-	1.5 ± 0.04	-	49.0 ± 1.32	3.0	28.0	40.7	27.3
C16:0-C16:0	-	3.6 ± 0.11	-	2.3 ± 0.07	7.5	4.1	0.9	1.8
C16:0-C18:1	-	9.5 ± 0.24	-	1.5 ± 0.04	9.6	8.1	7.0	4.1
C16:0-C18:0	-	0.7 ± 0.03	-	0.2 ± 0.01	76.0	3.3	5.4	0.1
C18:1-C18:1	-	5.0 ± 0.12	-	13.0 ± 0.31	7.5	57.7	31.4	5.5
C18:1-C18:0	-	0.9 ± 0.02	-	0.8 ± 0.02	10.3	45.0	49.8	0.4
C18:0-C18:0	-	0.2 ± 0.01	-	0.0 ± 0.00	3.7	9.4	19.5	0.2
C18:1-C20:0	-	<LOQ	-	<LOQ	0.4	<LOQ	2.1	<LOQ
C18:0-C20:0	-	<LOQ	-	<LOQ	0.8	<LOQ	1.1	<LOQ
**STEROL (mg/kg)**								
Cholesterol	5.9 ± 2.2	8.0 ± 2.0	13.4 ± 0.5	13.1 ± 1.1	<LOQ	10.3	6.7	8.5
Brassicasterol	7.5 ± 0.9	8.4 ± 1.1	526.5 ± 4.7	539.2 ± 56.0	<LOQ	355.4	285.9	243.9
Campesterol	113.3 ± 8.5	121.2 ± 7.3	1994.0 ± 31.2	1926.8 ± 178.9	<LOQ	1466.2	1199.2	935.4
Stigmasterol	47.5 ± 3.5	50.2 ± 4.2	117.1 ± 1.4	122.9 ± 2.9	71.0	87.1	109.0	25.0
Clerosterol	<LOQ	<LOQ	49.1 ± 1.7	51.2 ± 2.2	<LOQ	14.4	10.9	<LOQ
β-Sitosterol	271.4 ± 30.7	282.7 ± 20.2	2542.6 ± 24.8	2615.9 ± 236.1	348.2	1778.0	1446.8	1472.2
Δ-5-Avenasterol	7.4 ± 4.3	10.8 ± 0.6	155.5 ± 1.6	160.7 ± 11.6	868.8	152.3	132.7	129.1
Δ-5-24-Stigmastadienol	<LOQ	<LOQ	63.9 ± 0.0	64.9 ± 1.9	<LOQ	21.5	16.0	<LOQ

NCIE: not chemically interesterified; CIE: chemically interesterified; CIE 1: Shea 100%; CIE 2: mix Rapeseed (Raw/Hydrog 70/30); CIE 3: mix Rapeseed (Raw/Hydrog 60/40); CIE 4: mix Rapeseed/Palm 50/50; -: not detected.

## Data Availability

Data is available upon request.

## References

[1] Moreda-Piñeiro J., Moreda-Piñeiro A. (2019). Combined assisted extraction techniques as green sample pre-treatments in food analysis. TrAC Trends Anal. Chem..

[2] Moret S., Conchione C., Srbinovska A., Lucci P. (2019). Microwave-based technique for fast and reliable extraction of organic contaminants from food, with a special focus on hydrocarbon contaminants. Foods.

[3] Smith F.E., Arsenault E.A. (1996). Microwave-assisted sample preparation in analytical chemistry. Talanta.

[4] Gfrerer M., Lankmayr E. (2003). Microwave-assisted saponification for the determination of 16 polycyclic aromatic hydrocarbons from pumpkin seed oils. J. Sep. Sci..

[5] Hernández-Borges J., Rodríguez-Delgado M.A., García-Montelongo F.J. (2006). Optimization of the microwave-assisted saponification and extraction of organic pollutants from marine biota using experimental design and artificial neural networks. Chromatographia.

[6] Pena T., Pensado L., Casais C., Mejuto C., Phan-Tan-Luu R., Cela R. (2006). Optimization of a microwave-assisted extraction method for the analysis of polycyclic aromatic hydrocarbons from fish samples. J. Chromatogr. A.

[7] Navarro P., Cortazar E., Bartolomé L., Deusto M., Raposo J.C., Zuloaga O., Arana G., Etxebarria N. (2006). Comparison of solid phase extraction, saponification and gel permeation chromatography for the clean-up of microwave-assisted biological extracts in the analysis of polycyclic aromatic hydrocarbons. J. Chromatogr. A.

[8] Moret S., Scolaro M., Barp L., Purcaro G., Conte L.S. (2016). Microwave assisted saponification (MAS) followed by on-line liquid chromatography (LC)-gas chromatography (GC) for high-throughput and high-sensitivity determination of mineral oil in different cereal-based foodstuffs. Food Chem..

[9] Moret S., Purcaro G., Conte L.S. (2010). Polycyclic aromatic hydrocarbons (PAHs) levels in propolis and propolis-based dietary supplements from the Italian market. Food Chem..

[10] Akpambang V.O.E., Purcaro G., Lajide L., Amoo I.A., Conte L.S., Moret S. (2009). Determination of polycyclic aromatic hydrocarbons (PAHs) in commonly consumed Nigerian smoked/grilled fish and meat. Food Addit. Contam. Part A Chem. Anal. Control. Expo. Risk Assess..

[11] De Medina V.S., Priego-Capote F., Luque de Castro M.D. (2013). Comparison of saponification methods for characterization of the nonsaponifiable fraction of virgin olive oil. Eur. J. Lipid Sci. Technol..

[12] Ruiz-Gutiérrez V., Pérez-Camino M.C. (2000). Update on solid-phase extraction for the analysis of lipid classes and related compounds. J. Chromatogr. A.

[13] Gorassini A., Verardo G., Bortolomeazzi R. (2019). Polymeric reversed phase and small particle size silica gel solid phase extractions for rapid analysis of sterols and triterpene dialcohols in olive oils by GC-FID. Food Chem..

[14] Mathison B., Holstege D. (2013). A rapid method to determine sterol, erythrodiol, and uvaol concentrations in olive oil. J. Agric. Food Chem..

[15] Collison M.W. (2017). Official Methods and Recommended Practices of the AOCS.

[16] Paquot C. (1979). IUPAC Standard Methods for the Analysis of Oils, Fats and Derivatives.

[17] Moreau R.A., Whitaker B.D., Hicks K.B. (2002). Phytosterols, phytostanols, and their conjugates in foods: Structural diversity, quantitative analysis, and health-promoting uses. Prog. Lipid Res..

[18] Piironen V., Lindsay D.G., Miettinen T.A., Toivo J., Lampi A.M. (2000). Plant sterols: Biosynthesis, biological function and their importance to human nutrition. J. Sci. Food Agric..

[19] Azadmard-Damirchi S. (2010). Review of the use of phytosterols as a detection tool for adulteration of olive oil with hazelnut oil. Food Addit. Contam. Part A Chem. Anal. Control. Expo. Risk Assess..

[20] Lütjohann D. (2015). Methodological aspects of plant sterol and stanol measurement. J. AOAC Int..

[21] Ham B., Butler B., Thionville P. (2000). Evaluating the Isolation and Quantification of Sterols in Seed Oils by Solid-Phase Extraction and Capillary Gas—Liquid Chromatography Traditional methods of sterol fraction analysis involve saponification of. LCGC.

[22] Azadmard-Damirchi S., Dutta P.C. (2006). Novel solid-phase extraction method to separate 4-desmethyl-, 4-monomethyl-, and 4,4′-dimethylsterols in vegetable oils. J. Chromatogr. A.

[23] Zhu T.W., Weng H.T., Zhang X., Wu H., Li B. (2018). Mechanistic insight into the relationship between triacylglycerol and crystallization of lipase-catalyzed interesterified blend of palm stearin and vegetable oil. Food Chem..

[24] Verhe R., Van Hoed V., De Greyt W. Detection of alkyl ketones during chemical interesterification of lipids. Proceedings of the Paper Presented at 97th AOCS Annual Meeting & Expo.

[25] Mariani C., Bellan G. (2011). Individuazione di oli e grassi interesterificati con metodologie diverse. Riv. Ital. Delle Sostanze Grasse.

[26] Santoro V., Baiocchi C., Dal Bello F., Gastaldi D., Aigotti R., Zorzi M., Pellegrino A., Forte E., Romaniello F., Magni M. (2018). Formation of by-products during chemical interesterification of lipids. Detection and characterization of dialkyl ketones by non-aqueous reversed-phase liquid chromatography-high resolution mass spectrometry and gas chromatography-mass spectrometry. J. Chromatogr. A.

[27] Danthine S., De Clercq N., Dewettinck K., Gibon V. (2014). Monitoring batch lipase catalyzed interesterification of palm oil and fractions by differential scanning calorimetry. J. Therm. Anal. Calorim..

[28] De Clercq N., Danthine S., Nguyen M.T., Gibon V., Dewettinck K. (2012). Enzymatic interesterification of palm oil and fractions: Monitoring the degree of interesterification using different methods. JAOCS J. Am. Oil Chem. Soc..

[29] International Olive Council (IOC) (2017). Determination of the Composition and Content of Sterols and Triterpene Dialcohols by Capillary Column Gas Chromatography.

[30] Azadmard-Damirchi S., Dutta P.C. (2008). Stability of minor lipid components with emphasis on phytosterols during chemical interesterification of a blend of refined olive oil and palm stearin. JAOCS J. Am. Oil Chem. Soc..

[31] Gałuszka A., Migaszewski Z.M., Konieczka P., Namieśnik J. (2012). Analytical Eco-Scale for assessing the greenness of analytical procedures. TrAC Trends Anal. Chem..

